# (*E*)-4-Bromo-*N*-{(*E*)-3-[(4-bromo-2-methyl­phen­yl)imino]­butan-2-yl­idene}-2-methyl­aniline

**DOI:** 10.1107/S1600536812052087

**Published:** 2013-01-09

**Authors:** Jin-Li Yao, Xu Zhang, Hong-Yan Li, Jian-Qing Ye

**Affiliations:** a Key Laboratory of Environmental Materials and Engineering of Jiangsu Province, Yangzhou University, Yangzhou 225009, People’s Republic of China

## Abstract

The title compound, C_18_H_18_Br_2_N_2_, is centrosymmetric with the mid-point of the central C—C bond of the butyl group located on an inversion center. The terminal benzene ring is approximately perpendicular to the central butyl plane [dihedral angle = 71.9 (8)°]. No hydrogen bonding or aromatic stacking is observed in the crystal.

## Related literature

For applications of diimine-metal catalysts, see: Johnson *et al.* (1995 ▶); Killian *et al.* (1996 ▶); Popeney & Guan (2010 ▶); Popeney *et al.* (2011 ▶); Yuan *et al.* (2005 ▶). For a related structure, see: Zhang *et al.* (2013 ▶).
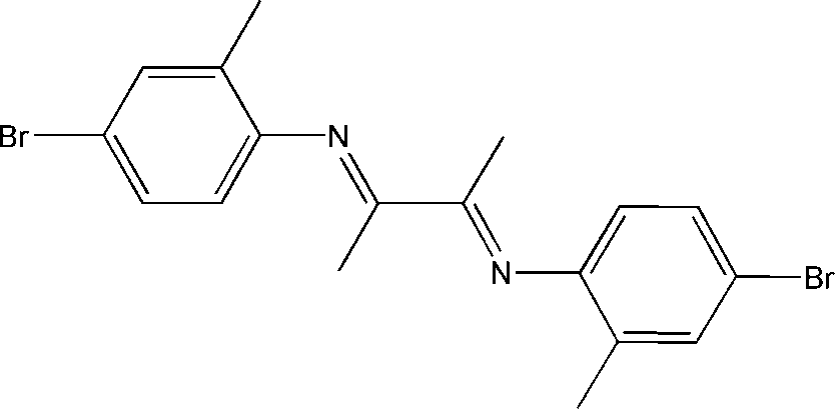


## Experimental

### 

#### Crystal data

C_18_H_18_Br_2_N_2_
*M* *_r_* = 422.16
Orthorhombic, 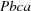

*a* = 13.625 (13) Å
*b* = 7.495 (7) Å
*c* = 17.029 (17) Å
*V* = 1739 (3) Å^3^
*Z* = 4
Mo *K*α radiation
μ = 4.66 mm^−1^
*T* = 293 K
0.21 × 0.20 × 0.15 mm

#### Data collection

Bruker APEXII CCD diffractometer
Absorption correction: multi-scan (*SADABS*; Bruker, 2001 ▶) *T*_min_ = 0.441, *T*_max_ = 0.542
6368 measured reflections
1541 independent reflections
847 reflections with *I* > 2σ(*I*)
*R*_int_ = 0.104

#### Refinement

*R*[*F* ^2^ > 2σ(*F* ^2^)] = 0.052
*wR*(*F* ^2^) = 0.113
*S* = 1.05
1541 reflections
103 parameters
H-atom parameters constrained
Δρ_max_ = 0.70 e Å^−3^
Δρ_min_ = −0.85 e Å^−3^

### 

Data collection: *APEX2* (Bruker, 2007 ▶); cell refinement: *SAINT* (Bruker, 2007 ▶); data reduction: *SAINT*; program(s) used to solve structure: *SHELXTL* (Sheldrick, 2008 ▶); program(s) used to refine structure: *SHELXTL*; molecular graphics: *SHELXTL*; software used to prepare material for publication: *SHELXTL*.

## Supplementary Material

Crystal structure: contains datablock(s) I, global. DOI: 10.1107/S1600536812052087/xu5666sup1.cif

Structure factors: contains datablock(s) I. DOI: 10.1107/S1600536812052087/xu5666Isup2.hkl

Supplementary material file. DOI: 10.1107/S1600536812052087/xu5666Isup3.cml

Additional supplementary materials:  crystallographic information; 3D view; checkCIF report

## Figures and Tables

**Fig. 1.**
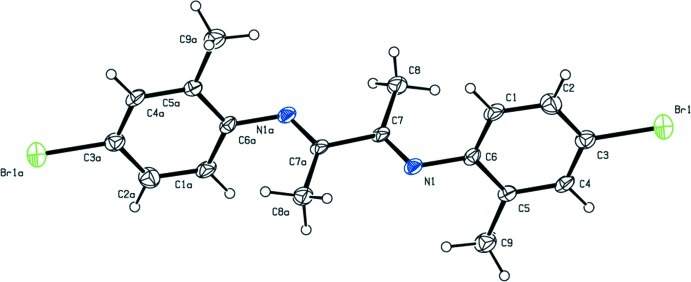
Molecular structure of the title compound, using 30% probability level ellipsoids.

## supplementary crystallographic information

### Comment

There is a considerable interest in the development of new late transition metal catalysts for the polymerization of α-olefins since Brookhart discovered highly active α-diimine nickel catalysts (Johnson *et al.*, 1995; Killian *et al.*, 1996). It is well known that the ligand structure had significant influence on the product properties and polymerization activities (Popeney & Guan, 2010; Popeney *et al.*, 2011; Yuan *et al.*, 2005).

In this study, we designed and synthesized the title compound as a bidentate ligand, and its molecular structure was characterized by X-ray diffraction. In the solid state, the ligand exhibits a -1 symmetry. The single bond of 1,4-diazabutadiene fragment is (E)-configured. The dihedral angle between the benzene ring and 1,4-diazabutadiene plane is 71.9 (8)°, similar to that found in a related compound (Zhang *et al.*, 2013). In the crystal packing, there is no hydrogen-bond between the molecules.

### Experimental

Formic acid (0.5 ml) was added to a stirred solution of 2,3-butanedione (0.103 g, 1.2 mmol) and 4-bromo-2-methylaniline (0.447 g, 2.4 mmol) in methanol (25 ml). The mixture was refluxed for 24 h, then cooled and the precipitate was separated by filtration. The solid was recrystallized from dichloromethane/cyclohexane (*v*/*v* = 6:1), washed with cold ethanol and dried under vacuum to give the title ligand 0.37 g (87%). Anal. Calcd. for C_18_H_18_Br_2_N_2_: C, 51.21; H, 4.30; N, 6.64. Found: C, 51.18; H, 4.29; N, 6.68. Crystals suitable for X-ray structure determination were grown from a solution of the title compound in a mixture of cyclohexane/dichloromethane (1:4, *v*/*v*).

### Refinement

All hydrogen atoms were placed in calculated positions with C—H distances of 0.93 and 0.96 Å for aryl and methyl type H-atoms. They were included in the refinement in a riding model approximation, respectively. The H-atoms were assigned *U*_iso_ = 1.2 times *U*_eq_ of the aryl C atoms and 1.5 times *U*_eq_ of the methyl C atoms.

### Crystal data

**Table:**

C_18_H_18_Br_2_N_2_	*F*(000) = 840
*M**_r_* = 422.16	*D*_x_ = 1.612 Mg m^−^^3^
Orthorhombic, *P**b**c**a*	Mo *K*α radiation, λ = 0.71073 Å
Hall symbol: -P 2ac 2ab	Cell parameters from 951 reflections
*a* = 13.625 (13) Å	θ = 2.8–20.3°
*b* = 7.495 (7) Å	µ = 4.66 mm^−^^1^
*c* = 17.029 (17) Å	*T* = 293 K
*V* = 1739 (3) Å^3^	Block, yellow
*Z* = 4	0.21 × 0.20 × 0.15 mm

### Data collection

**Table:**

Bruker APEXII CCD diffractometer	1541 independent reflections
Radiation source: fine-focus sealed tube	847 reflections with *I* > 2σ(*I*)
Graphite monochromator	*R*_int_ = 0.104
φ and ω scans	θ_max_ = 25.3°, θ_min_ = 2.8°
Absorption correction: multi-scan (*SADABS*; Bruker, 2001)	*h* = −15→15
*T*_min_ = 0.441, *T*_max_ = 0.542	*k* = −9→5
6368 measured reflections	*l* = −20→18

### Refinement

**Table:**

Refinement on *F*^2^	Secondary atom site location: difference Fourier map
Least-squares matrix: full	Hydrogen site location: inferred from neighbouring sites
*R*[*F*^2^ > 2σ(*F*^2^)] = 0.052	H-atom parameters constrained
*wR*(*F*^2^) = 0.113	*w* = 1/[σ^2^(*F*_o_^2^) + (0.0106*P*)^2^ + 5.9013*P*] where *P* = (*F*_o_^2^ + 2*F*_c_^2^)/3
*S* = 1.05	(Δ/σ)_max_ < 0.001
1541 reflections	Δρ_max_ = 0.70 e Å^−^^3^
103 parameters	Δρ_min_ = −0.85 e Å^−^^3^
0 restraints	Extinction correction: *SHELXTL* (Sheldrick, 2008), Fc^*^=kFc[1+0.001xFc^2^λ^3^/sin(2θ)]^-1/4^
Primary atom site location: structure-invariant direct methods	Extinction coefficient: 0.0038 (5)

### Special details

**Table:**

Geometry. All e.s.d.'s (except the e.s.d. in the dihedral angle between two l.s. planes) are estimated using the full covariance matrix. The cell e.s.d.'s are taken into account individually in the estimation of e.s.d.'s in distances, angles and torsion angles; correlations between e.s.d.'s in cell parameters are only used when they are defined by crystal symmetry. An approximate (isotropic) treatment of cell e.s.d.'s is used for estimating e.s.d.'s involving l.s. planes.
Refinement. Refinement of *F*^2^ against ALL reflections. The weighted *R*-factor *wR* and goodness of fit *S* are based on *F*^2^, conventional *R*-factors *R* are based on *F*, with *F* set to zero for negative *F*^2^. The threshold expression of *F*^2^ > σ(*F*^2^) is used only for calculating *R*-factors(gt) *etc*. and is not relevant to the choice of reflections for refinement. *R*-factors based on *F*^2^ are statistically about twice as large as those based on *F*, and *R*– factors based on ALL data will be even larger.

### Fractional atomic coordinates and isotropic or equivalent isotropic displacement parameters (Å^2^)

**Table:**

	*x*	*y*	*z*	*U*_iso_*/*U*_eq_	
Br1	0.30195 (6)	0.23158 (11)	0.40014 (4)	0.0631 (4)	
C1	0.4677 (5)	0.4420 (8)	0.2188 (4)	0.0398 (17)	
H1	0.5330	0.4728	0.2107	0.048*	
C2	0.4386 (5)	0.3772 (9)	0.2914 (4)	0.0444 (19)	
H2	0.4836	0.3653	0.3321	0.053*	
C3	0.3421 (5)	0.3308 (8)	0.3021 (4)	0.0374 (17)	
C4	0.2742 (5)	0.3508 (7)	0.2429 (4)	0.0341 (16)	
H4	0.2092	0.3188	0.2518	0.041*	
C5	0.3017 (5)	0.4189 (7)	0.1692 (3)	0.0277 (14)	
C6	0.3998 (4)	0.4617 (7)	0.1574 (4)	0.0299 (15)	
C7	0.4845 (4)	0.4520 (8)	0.0370 (4)	0.0307 (15)	
C8	0.5198 (5)	0.2627 (8)	0.0474 (4)	0.0451 (17)	
H8A	0.4765	0.2006	0.0825	0.068*	
H8B	0.5850	0.2636	0.0689	0.068*	
H8C	0.5205	0.2035	−0.0026	0.068*	
C9	0.2258 (4)	0.4423 (8)	0.1053 (4)	0.0452 (18)	
H9A	0.2541	0.5074	0.0623	0.068*	
H9B	0.1706	0.5071	0.1257	0.068*	
H9C	0.2045	0.3273	0.0872	0.068*	
N1	0.4299 (4)	0.5380 (6)	0.0839 (3)	0.0331 (13)	

### Atomic displacement parameters (Å^2^)

**Table:**

	*U*^11^	*U*^22^	*U*^33^	*U*^12^	*U*^13^	*U*^23^
Br1	0.0619 (6)	0.0768 (6)	0.0505 (5)	0.0068 (5)	0.0143 (4)	0.0208 (5)
C1	0.017 (4)	0.044 (4)	0.058 (5)	−0.003 (3)	0.004 (3)	0.006 (4)
C2	0.040 (5)	0.048 (5)	0.045 (4)	−0.005 (4)	−0.007 (3)	0.004 (4)
C3	0.033 (4)	0.031 (4)	0.048 (4)	0.001 (3)	0.003 (4)	0.000 (3)
C4	0.021 (4)	0.030 (3)	0.051 (4)	−0.001 (3)	0.009 (3)	−0.002 (3)
C5	0.024 (4)	0.019 (3)	0.041 (4)	−0.001 (3)	0.002 (3)	−0.001 (3)
C6	0.027 (4)	0.021 (3)	0.042 (4)	0.001 (3)	0.012 (3)	0.000 (3)
C7	0.012 (3)	0.028 (4)	0.052 (4)	−0.005 (3)	0.004 (3)	0.008 (3)
C8	0.043 (4)	0.033 (4)	0.060 (4)	0.012 (4)	0.015 (4)	0.012 (3)
C9	0.033 (5)	0.043 (4)	0.059 (5)	−0.002 (3)	0.003 (4)	0.003 (4)
N1	0.021 (3)	0.030 (3)	0.048 (4)	0.000 (3)	0.006 (3)	0.006 (3)

### Geometric parameters (Å, º)

**Table:**

Br1—C3	1.908 (7)	C6—N1	1.436 (7)
C1—C2	1.386 (9)	C7—N1	1.268 (7)
C1—C6	1.403 (8)	C7—C8	1.509 (8)
C1—H1	0.9300	C7—C7^i^	1.510 (11)
C2—C3	1.372 (9)	C8—H8A	0.9600
C2—H2	0.9300	C8—H8B	0.9600
C3—C4	1.375 (9)	C8—H8C	0.9600
C4—C5	1.406 (8)	C9—H9A	0.9600
C4—H4	0.9300	C9—H9B	0.9600
C5—C6	1.390 (8)	C9—H9C	0.9600
C5—C9	1.512 (8)		
			
C2—C1—C6	120.8 (6)	C1—C6—N1	120.2 (5)
C2—C1—H1	119.6	N1—C7—C8	126.2 (5)
C6—C1—H1	119.6	N1—C7—C7^i^	116.6 (7)
C3—C2—C1	118.8 (6)	C8—C7—C7^i^	117.2 (7)
C3—C2—H2	120.6	C7—C8—H8A	109.5
C1—C2—H2	120.6	C7—C8—H8B	109.5
C2—C3—C4	121.3 (6)	H8A—C8—H8B	109.5
C2—C3—Br1	119.3 (5)	C7—C8—H8C	109.5
C4—C3—Br1	119.4 (5)	H8A—C8—H8C	109.5
C3—C4—C5	120.9 (6)	H8B—C8—H8C	109.5
C3—C4—H4	119.5	C5—C9—H9A	109.5
C5—C4—H4	119.5	C5—C9—H9B	109.5
C6—C5—C4	118.0 (6)	H9A—C9—H9B	109.5
C6—C5—C9	121.8 (6)	C5—C9—H9C	109.5
C4—C5—C9	120.2 (6)	H9A—C9—H9C	109.5
C5—C6—C1	120.1 (6)	H9B—C9—H9C	109.5
C5—C6—N1	119.5 (6)	C7—N1—C6	120.9 (5)
			
C6—C1—C2—C3	0.6 (10)	C4—C5—C6—N1	−177.6 (5)
C1—C2—C3—C4	−1.3 (10)	C9—C5—C6—N1	2.9 (8)
C1—C2—C3—Br1	177.3 (5)	C2—C1—C6—C5	1.1 (9)
C2—C3—C4—C5	0.3 (9)	C2—C1—C6—N1	176.6 (5)
Br1—C3—C4—C5	−178.3 (4)	C8—C7—N1—C6	3.9 (10)
C3—C4—C5—C6	1.4 (8)	C7^i^—C7—N1—C6	−177.3 (6)
C3—C4—C5—C9	−179.2 (6)	C5—C6—N1—C7	−112.5 (7)
C4—C5—C6—C1	−2.0 (8)	C1—C6—N1—C7	71.9 (8)
C9—C5—C6—C1	178.5 (5)		

Symmetry code: (i) −*x*+1, −*y*+1, −*z*.
